# The Thomas Force Young Investigator Award

**DOI:** 10.1016/j.jacbts.2021.10.006

**Published:** 2021-11-22

**Authors:** Douglas L. Mann

On behalf of the Editors, I am pleased to announce that beginning this year, *JACC: Basic to Translational Science* will present the recipient of the most outstanding research paper by a primary author at the early stage of their career, with the Thomas Force Young Investigator Award. This award will also recognize the primary mentor for the young investigator. The Force award will be announced at the annual meeting of the American College of Cardiology, and the awardee and their mentor featured in the May 2022 issue of *JACC: Basic to Translational Science.*

**Figure 1:**
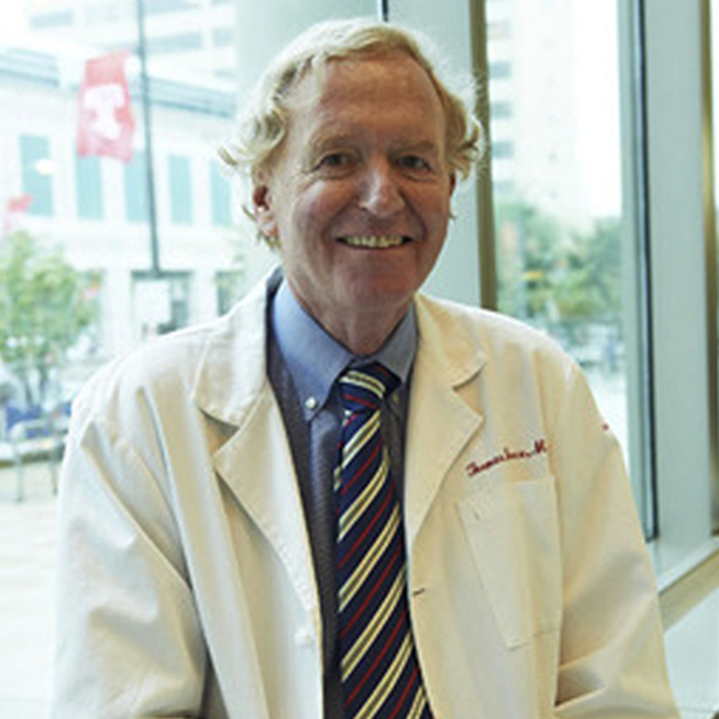
Thomas L. Force, MD (1951-2020)

Dr Thomas Force (Figure 1) was one of the founding associate editors for *JACC: Basic to Translational Science*. He remained on the editorial board until he retired in 2016. The entire academic community mourned his passing on November 30th, 2020.

Tom was many things to many people, all which are captured in a wonderful memorial article about Tom’s life and career published in the March 2021 issue of *JACC: CardioOncology* (1). To me, Tom was a good friend and close colleague who cared as deeply as I do about the integrity of translational science. He was always more concerned about getting the science right than he was getting out in front of the news media to proclaim the latest cure for heart failure. When I was thinking about whom I should ask to become a founding associate editor for *JACC: Basic to Translational Science*, Tom was at the top of my list. As I began to explain to Tom why I thought that it was important to create a literary home for scientists whose work bridged both basic and clinical science, he cut me short and said, “I’m in….just tell me what you need me to do.” As I said, Tom cared deeply about the integrity of the scientific process. He could also be laconic at times, especially when I rambled too much.

Tom’s brilliant research career has been detailed by others, and I will not try to repeat his scientific journey here (1). Rather, what I want to focus on, which is directly relevant to this award, is that Tom was an incredible mentor who stimulated countless trainees to pursue careers in science. His breadth of mentoring skills allowed him to train MDs, MD/PhDs, and PhDs. He was exceedingly generous with his time. He created opportunities for his mentees, often times giving up the literary spotlight (ie, first or last author) so that his trainees would get the credit. When a trainee left his lab, he continued to advocate for them throughout the arc of their career. Most importantly, Tom made science fun. One could not help but become energized by watching Tom discuss science with trainees or colleagues. He was unique in this regard, because he always kept the focus on the science, without ever drawing attention to himself. I know that have become a better mentor by watching how Tom interacted with his trainees.

We lost a dear friend, an amazing person, a brilliant scientist, and an incredible mentor on November 30th, 2020. My sincere hope is that we will keep Tom’s memory alive each year by recognizing the next generation of investigators and their mentors by creating the Thomas Force Young Investigator Award.
